# Photoacoustic imaging of fresh human surgically and endoscopically resected gastrointestinal specimens

**DOI:** 10.1002/deo2.28

**Published:** 2021-08-16

**Authors:** Hiroaki Ikematsu, Miya Ishihara, Shinpei Okawa, Tatsunori Minamide, Tomohiro Mitsui, Takeshi Kuwata, Masaaki Ito, Takahiro Kinoshita, Takeo Fujita, Tomonori Yano, Toshihiko Omori, Satoshi Ozawa, Dai Murakoshi, Kaku Irisawa, Atsushi Ochiai

**Affiliations:** ^1^ Division of Science and Technology for Endoscopy Exploratory Oncology Research and Clinical Trial Center National Cancer Center Chiba Japan; ^2^ Department of Gastroenterology and Endoscopy National Cancer Center Hospital East Chiba Japan; ^3^ Department of Medical Engineering National Defense Medical College Saitama Japan; ^4^ Department of Pathology and Clinical Laboratories National Cancer Center Hospital East Chiba Japan; ^5^ Department of Colorectal Surgery National Cancer Center Hospital East Chiba Japan; ^6^ Department of Gastric Surgery National Cancer Center Hospital East Chiba Japan; ^7^ Department of Esophageal Surgery National Cancer Center Hospital East Chiba Japan; ^8^ Medical Systems Research & Development Center Research & Development Management Headquarters FUJIFILM Corporation Kanagawa Japan; ^9^ Exploratory Oncology Research and Clinical Trial Center National Cancer Center Chiba Japan

**Keywords:** deep vessel network, gastrointestinal lesions, optoacoustic imaging

## Abstract

**Objective:**

Photoacoustic (PA) imaging is a novel noninvasive technique that offers high‐contrast tomographic imaging with ultrasound‐like resolution at depths of centimeters, enabling visualization of deep small vessels. The aim of this pilot study was to survey the characteristics of deep vessel networks in the mucosa of neoplastic gastrointestinal (GI) lesions using PA imaging.

**Methods:**

Specimens of patients who had undergone surgical and endoscopic resection for GI lesions were included in this study. The PA/ultrasound imaging system for clinical research is characterized by a technology that can superimpose a PA image over an ultrasound image. Three‐dimensional PA images were acquired for the resected specimen before fixation. The stomach and colon of live pigs were incised, and the walls were scanned from the mucosa.

**Results:**

A total of 32 specimens (nine esophageal, 12 gastric, 11 colorectal) were scanned. The pathological diagnoses were adenomas (*n* = 2), intramucosal cancers (*n* = 14), and invasive cancers (*n* = 16). The deep vessel networks of all lesions could be visualized. In the intramucosal lesions, the deep vessel network was similar to that of a normal tissue. In invasive cancers, the thick and prominent vessel network was visible in the surface layer of esophageal cancers, infiltrated area of gastric cancers, and surface layer and infiltrated area of colorectal cancers. In the images of living pigs, visualizing the vascular network deeper than the submucosa in both the stomach and large intestine was possible.

**Conclusion:**

Our study confirmed that the deep vessel networks of neoplastic GI lesions were visible by PA imaging.

## INTRODUCTION

With the development of endoscopic imaging, it has become possible to observe high‐quality images by simultaneously employing various modalities, such as image enhancement endoscopy (IEE) observation, magnifying endoscopic observation, and endoscopic ultrasound (EUS) observation.

IEE and magnifying endoscopic observation can be used to observe in detail the capillaries and pit pattern on the surface of gastrointestinal (GI) lesions, enabling the prediction of a pathological diagnosis without biopsy. Furthermore, diagnosis by these observations is reportedly useful for qualitative and depth diagnoses related to treatment decisions for early lesions.^1^, ^2^, ^3^, ^4^ However, all these diagnoses infer deep lesion characteristics from the findings on the lesion surface, which is not sufficient for treatment.^5^, ^6^, ^7^, ^8^

Although EUS can obtain deep information, it has been reported that its diagnostic ability is not different from that of magnifying endoscopic diagnosis.^9^ In recent years, optical coherence tomography (OCT) has also been used in the GI tract, which enables deep diagnosis with clearer image quality than that obtained using EUS.^10^ However, the visualization is limited to a depth of 2000 μm owing to effects such as light scattering. Therefore, there are limitations in the diagnosis of certain GI lesions, particularly in polypoid lesions that have a low diagnostic ability in the surface layer of the lesion.

In the Doppler mode of EUS, the blood flow in the deep and thick blood vessels can be evaluated; however, it is difficult to recognize the thin blood vessels in the shallow layer owing to the blood vessel diameter.

In early GI lesions, recognizing the blood vessels in the GI wall is necessary for elucidation of the pathological condition and can be expected to be useful for invasion diagnosis. Visualization of the blood vessels in the shallow layer of GI lesions is expected to lead to accurate deep diagnosis and elucidation of the vessel network.

Photoacoustic (PA) imaging offers a high‐contrast tomographic image noninvasively with a US‐like resolution. Pulsed light meeting the laser safety standards applied to living tissue generates US referred to as PA wave depending on the level of optical absorption by substances in the tissue. Especially, the near‐infrared (NIR) light excites the PA waves from hemoglobin (Hb), which is the main absorber of NIR light in the living body. Then the PA image of the Hb distribution is reconstructed from the PA waves detected by a US transducer available for clinical use. The imaging depth is approximately up to 20 mm which depends on the penetration depth of light and detectability of the US transducer. It can be possible to quantify the concentrations of oxidized/reduced Hb by multispectral PA imaging, which has the potential to visualize and quantify deep vessel networks without injection of a contrast agent or exposure to X‐rays. By taking advantage of the noninvasive nature of the PA imaging, clinical PA imaging has been reported in a wide range,^11^, ^12^ including prostate cancer,^13^ skin cancer,^14^, ^15^ cerebral blood flow oxygenation disorder, intracranial hematoma,^16^, ^17^, thyroid cancer,^18^, ^19^, ^20^ inflammatory arthritis,^21^, ^22^ and breast cancer.^23^, ^24^ However, most PA imaging studies of the GI walls used animal models.^25^ Although PA imaging in Crohn's disease has been reported,^26^, ^27^ there is a dearth of research on the use of this method for specimens collected from human tissues, including cancer tissues.

The deep vessel network characteristics obtained using PA imaging may be useful for depth diagnosis and elucidation of the vascular network of GI cancer, but there are few reports on PA imaging of GI cancer.^28^, ^29^

The aim of this pilot study was to investigate the possibility of imaging deep vessel networks under the mucosa of neoplastic GI lesions using PA imaging.

## METHODS

### Study design and approval

The study was a single‐center prospective observational trial and animal experimentation. The protocol was approved by the institutional review board of the National Cancer Center (approval number: 2018–140) and the Committee for Ethics of Animal Experimentation of the National Cancer Center (IVT20–33). All participants provided written informed consent. The study was carried out in accordance with the World Medical Association Helsinki Declaration.

### Subjects

The specimens of patients who consented to undergo surgical and endoscopic resection for esophageal, gastric, and colorectal cancer, and adenoma at the National Cancer Center Hospital East, from November 2018 to August 2020 were included in this study.

In order to confirm the blood vessel image in real‐time, living miniature pigs were used. Laboratory animal experiments were carried out at the IVTeC Intervention Technical Center (Narita, Japan).

### Photoacoustic/US echo imaging system

**FIGURE 1:**
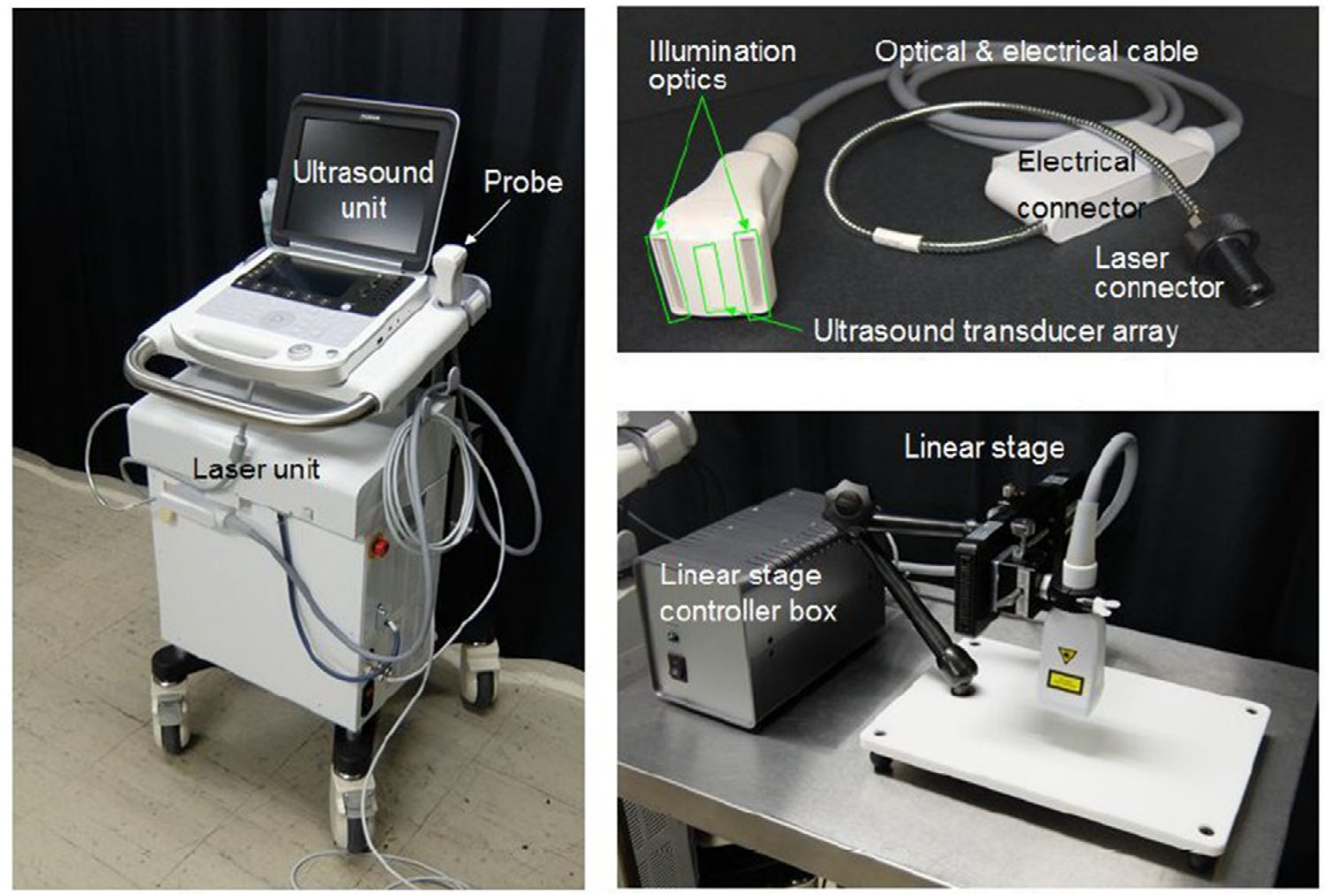
Photoacoustic (PA) image diagnostic device. The PC‐like user interface/ ultrasound imaging unit is mounted on the laser unit with the footswitch to trigger the pulsed laser lights on the floor (left), and the scanning unit with the linear photoacoustic probe attached to the motorized linear stage (center) and the PC for image processing after the PA image acquisitions (right) are placed on the desk. (A) Photoacoustic system, (B) photoacoustic probe, and (C) linear stage scanner for 3D image acquisition

Our team built a PA/US echo imaging system for clinical research (Fujifilm Co, Tokyo, Japan) (Figure 1).^30^ The system had an Alexandrite laser unit as the light source to excite the PA wave. The laser unit was operated with a wavelength of 750 nm, pulse width of 50 ± 10 ns, and a repetition rate of 10 Hz. The pulsed laser light was introduced into the hand‐held type PA linear probe via optical fiber bundles, and it illuminated the surface of the specimen from the diffusive optical windows placed along both sides of the linear US transducer array to detect the PA wave. The linear transducer array with a central frequency of 9 MHz had 128 elements lined with a space of 0.2 mm. By using the linear transducer array, the PA linear probe also can obtain the conventional high‐quality B‐mode US image simultaneously. This system is mainly characterized by a technology that can superimpose a PA image over a B‐mode US image.^30^ The real‐time simultaneous acquisitions of the PA and B‐mode images enabled the co‐registration of the images without a position gap. The co‐registered image makes it possible to precisely observe the blood vessels in the GI tract with a normal layer structure and abnormal lesions. In addition, the system is compact, ensuring the safety of the laser output with the fluence being much smaller than the maximum permissible exposure of 25.2 mJ/cm^2^ at the contact surface to meet the laser safety standards IEC60825‐1 (2007) (JIS C 6802(2011)) and to avoid damaging the specimen, and enabling the construction of a 3D image forming a sequence of 2D tomographic PA images.

### Measurement

The PA linear probe was attached to a scanning unit connected with a motorized linear stage (SGSP20‐85; SIGMAKOKI Co., Ltd., Tokyo, Japan) to scan the surface of the specimen on the table. The scanning unit is constructed to enable the adjustment of the scanning axis with a flexible arm so that the PA probe scans are parallel to the table.^31^ By scanning the PA probe with 0.2 mm steps, the sequence of 2D PA tomographic images with B‐mode US image of the specimen was acquired immediately after endoscopic and surgical resection. A US gel was used on both the mucosal and serosal sides of the specimen to match the acoustic impedance by filling the space between the PA linear probe and the specimen and to suppress light scattering at the surface of the specimen.

In the first few samples, the normal tissue was scanned to set up the scanner and to determine the optimal settings in the digestive tract. The lesions were then scanned under these conditions and continuously imaged.

Animal experiments were performed in accordance with the relevant guidelines and regulations. Live pigs were anesthetized, the stomachs and colons were incised while the animals were alive, and the walls were scanned from the mucosa. After completion of the experiment, the pigs were euthanized.

### Pathological evaluation

After the scan, the resected specimens were quickly fixed in formalin solution and sent to the pathology laboratory and examined histologically using hematoxylin and eosin staining. Histological diagnosis was performed by two independent pathologists blinded to the clinical information. Pathological results were defined in accordance with the criteria of the World Health Organization.^32^ The scanned images corresponded one‐to‐one with the macro image of the specimen and the pathological image to evaluate the vessel network.

### Outcomes

The PA images were compared to the pathological specimen of the part consistent with the scanned images and converted to 3D images by custom‐made software (USPAI; Fujifilm Co). The maximum intensity projections of the PA image were also constructed to examine the whole vessel distribution by the software. The 3D PA image superimposed on the 3D US image was generated from the image stacks by the image processing and analysis software ImageJ.^33^ The color scale of the PA images was replaced to indicate the depths from the probe by using MATLAB.

The primary endpoint was visualization of the vessel network deeper than the submucosa of GI lesions.

The secondary endpoint was the difference in deep vessel networks between invasive and noninvasive cancers and among each GI organ.

## RESULTS

A total of 32 patients with nine esophageal, 12 gastric, and 11 colorectal lesions were recruited (Table 1). A total of 16 surgical treatments and 16 endoscopic resections were performed. Pathological diagnosis included two adenomas, 14 intramucosal carcinomas, seven submucosal carcinomas, and nine T2–4 carcinomas. All cases were able to be scanned; however, in three advanced colorectal cancers, deep vessels were not well visualized due to the influence of fatty tissue. Therefore, they were excluded from this study.

**TABLE 1:** Background data of the specimen (*n* = 32)

	**Esophagus**	**Stomach**	**Colon and rectum**
*Resected method*			
Endoscopy	6	6	4
Surgery	3	6	7
*Depth*			
Mucosal layer (includes adenoma)	5	7	4
Submucosal layer	2	3	2
Deeper than the muscle layer	2	2	5

**FIGURE 2:**
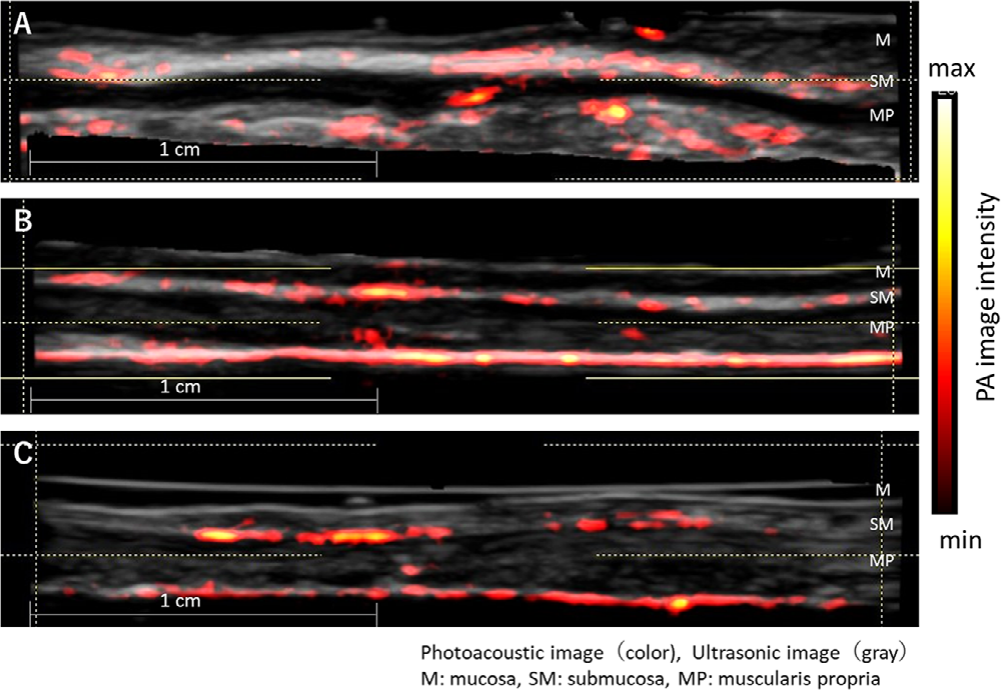
Photoacoustic (PA) image of normal tissue. PA image is superimposed on gray‐scaled B‐mode ultrasound (US) image. The color bar indicates the photoacoustic image intensity. (A) Esophagus, (B) stomach, and (C) rectum

In a normal tissue scan of one case each of esophagus, stomach, and rectum tissue, the deep vessel network was visible, but the blood vessels in the mucosal layer were not visible (Figure 2).

**FIGURE 3:**
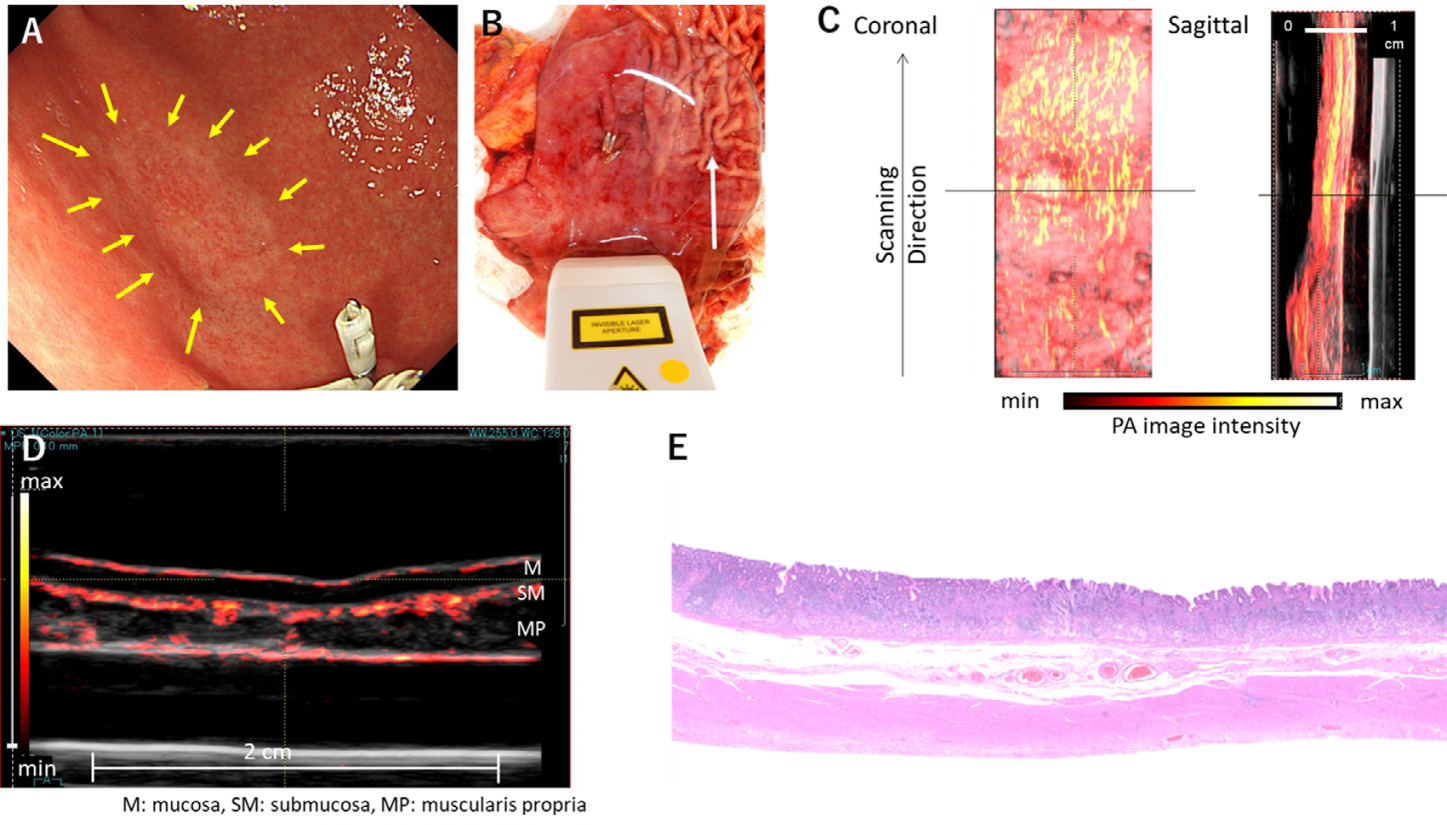
Gastric intramucosal cancer (signet ring cell carcinoma). Photoacoustic (PA) images are superimposed on the gray‐scaled B‐mode ultrasound (US) images in (C) and (D). The color bars indicate the PA image intensity. The maximum intensity projections of the 3D PA image onto the mucosal surface (coronal) and the longitudinal cross‐section (sagittal) of the specimen are shown in (C). The cross‐sectional image at the position indicated by the horizontal solid line in (C) is shown in (D). (A) Endoscopic image (20 mm, 0–IIc lesion), (B) scan direction, (C) whole scan coronal and sagittal images, (D) photoacoustic image of the lesion center, and (E) pathological image of the same site as the photoacoustic image

**FIGURE 4:**
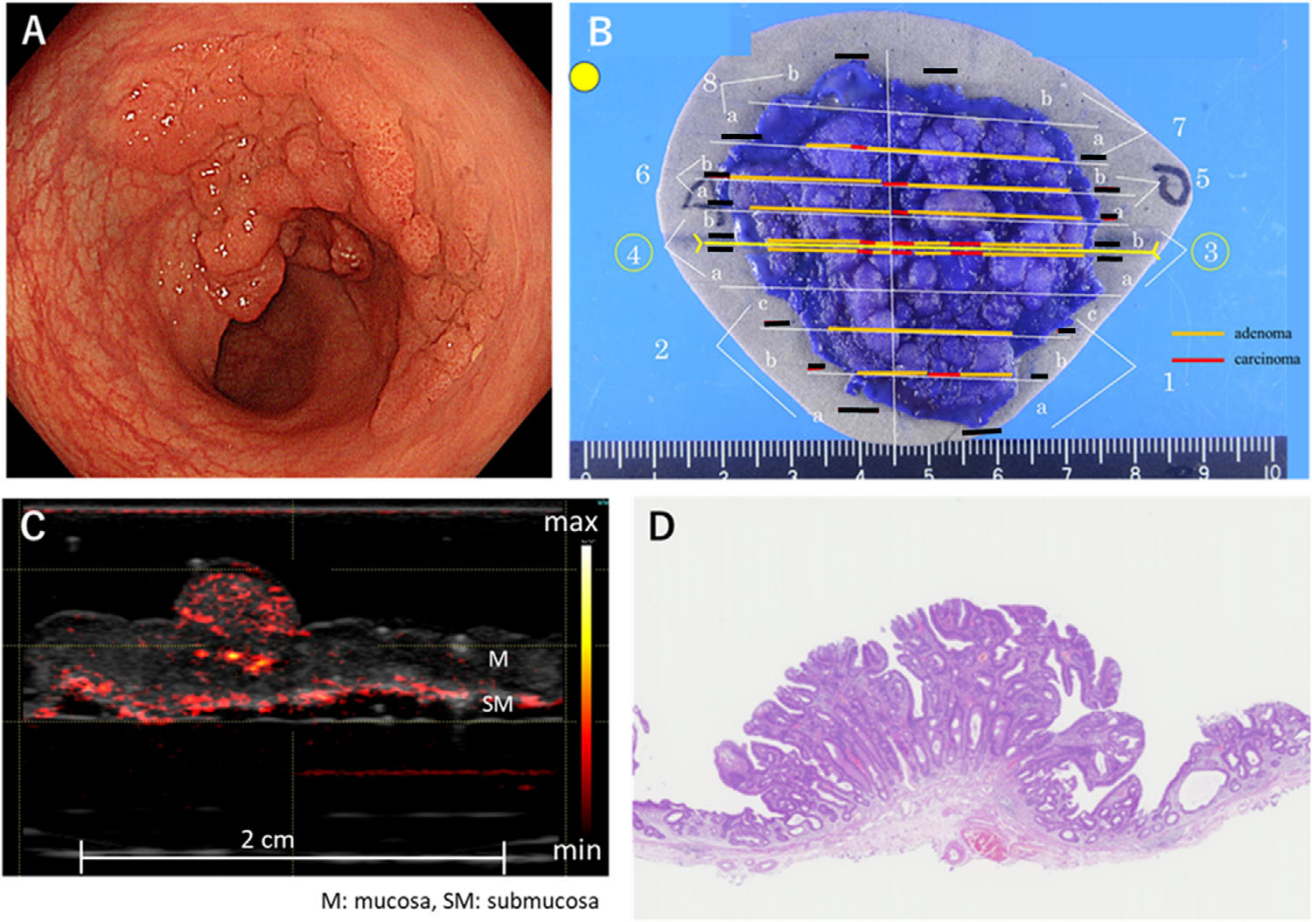
Cancer in adenoma in the sigmoid colon. Photoacoustic (PA) image is superimposed on gray‐scaled B‐mode US image in (C). The color bar indicates the PA image intensity. (A) Endoscopic image (50 mm, 0–Is+IIa lesion), (B) photograph of the resected lesion specimen, (C) photoacoustic image of the lesion center, and (D) pathological image of the same site as the photoacoustic image

In all of the specimens, the vessel networks in multiple‐layered normal and lesional tissues at a depth up to several millimeters could be visualized. In intramucosal lesions, the vessel network was similar to that of the normal tissue (Figure 3). Although the blood vessels of the mucosal layer were found in the elevated nodules of the colon lesions, the pathological diagnosis was cancer in adenoma and was not associated with the cancer component (Figure 4).

**FIGURE 5:**
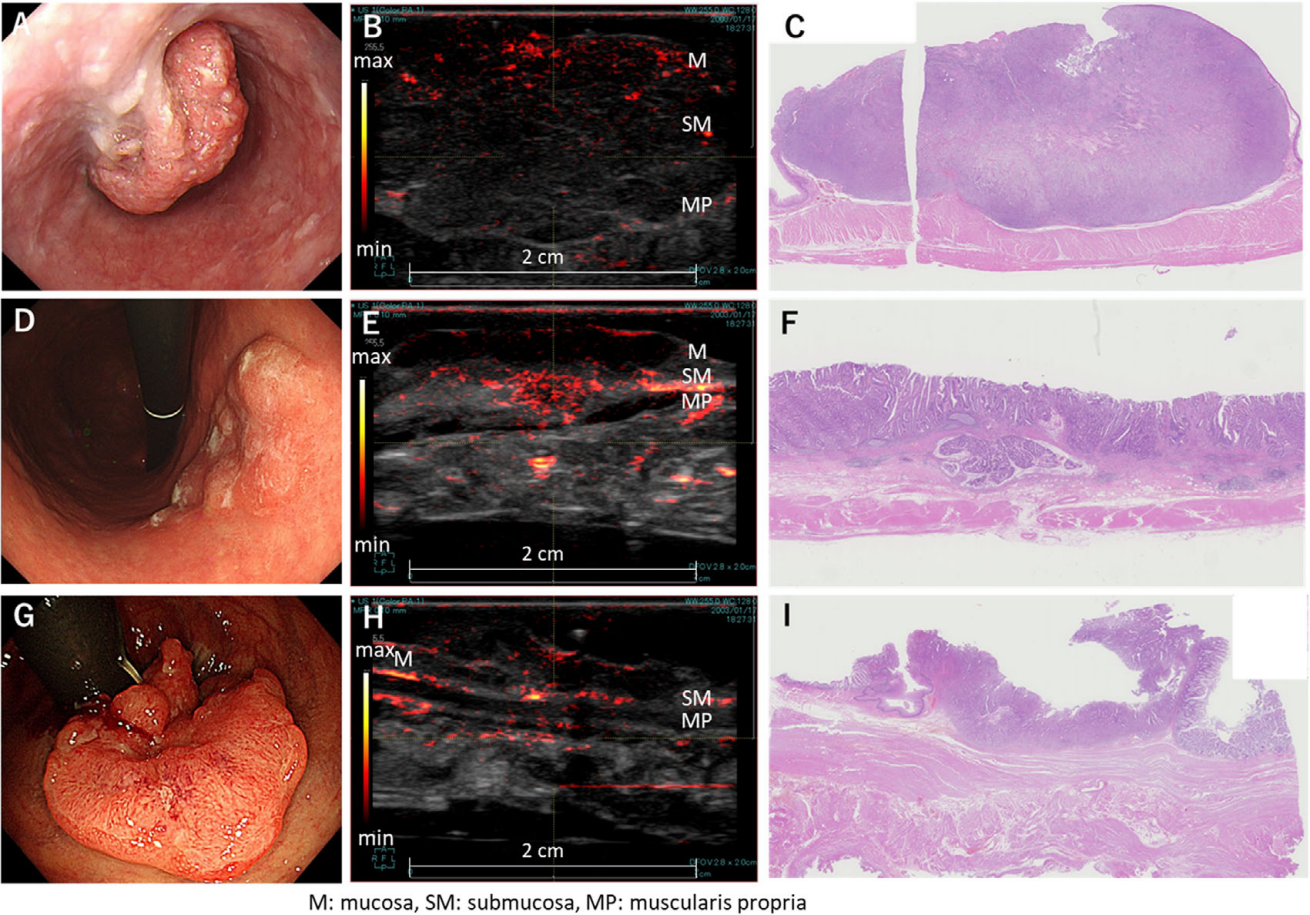
Photoacoustic (PA) image of T1 gastrointestinal cancer. PA images are superimposed on gray‐scaled B‐mode ultrasound (US) images in (B), (E), and (H). The color bars indicate the PA image intensity. (A) Esophagus, 30 mm, Is, pSM3, (B) photoacoustic image of the deepest invaded area of cancer, (C) pathological image of the same site as the photoacoustic image, (D) stomach, 40 mm, IIa, pT1b, (E) photoacoustic image of the deepest invaded area of cancer, (F) pathological image of the same site as the photoacoustic image, (G) rectum, 25 mm, IIa+IIc, pT1b, (H) photoacoustic image of the deepest invaded area of cancer, and (I) pathological image of the same site as the photoacoustic image

**FIGURE 6:**
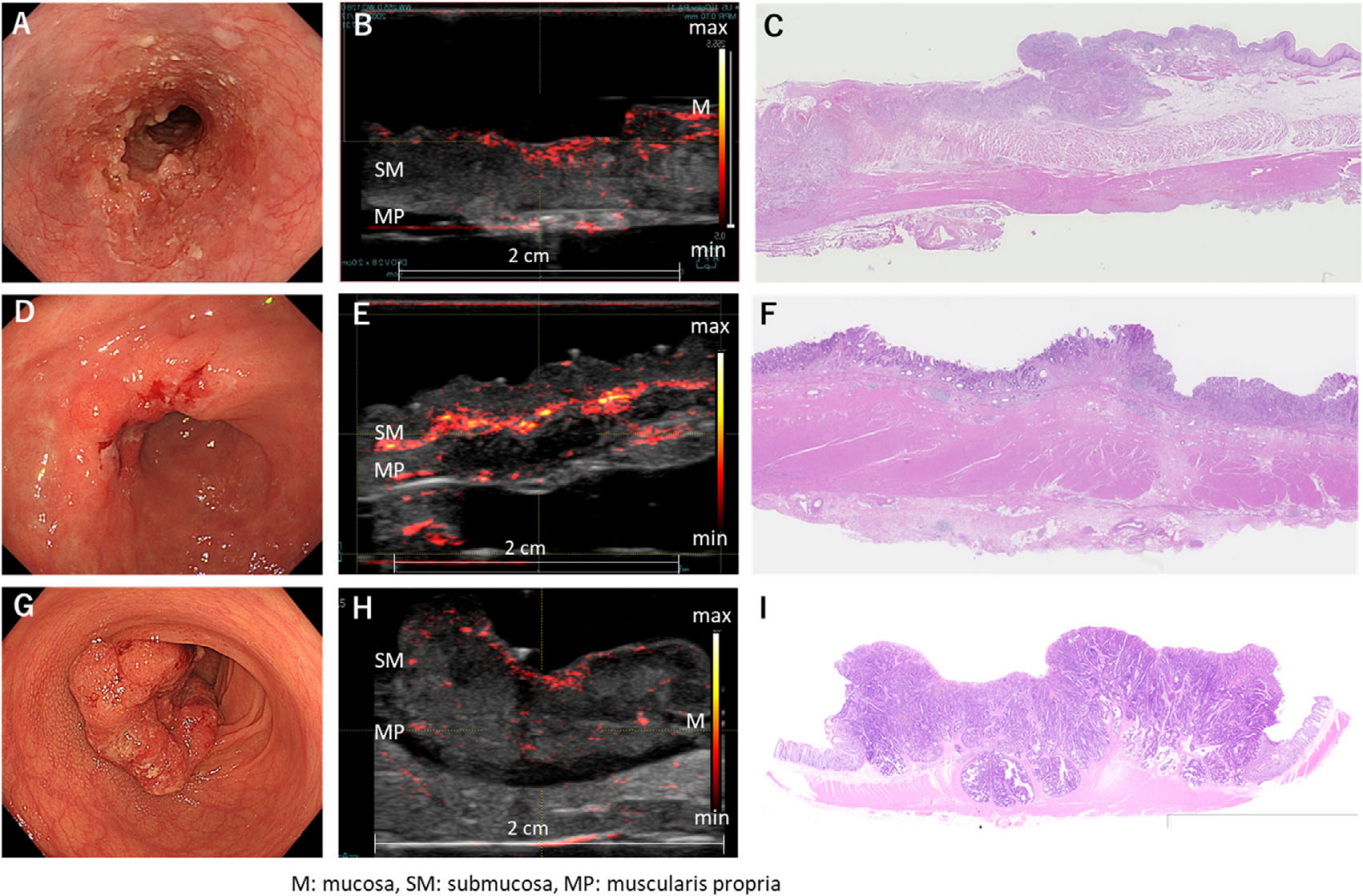
Photoacoustic (PA) image of advanced gastrointestinal cancer. PA images are superimposed on gray‐scaled B‐mode US images in (B), (E), and (H). The color bars indicate the PA image intensity. (A) Esophagus, Type2, pT3, (B) photoacoustic image from the edge to the center of the lesion, (C) pathological image of the same site as the photoacoustic image, (D) stomach, Type2, pT3, (E) photoacoustic image from the edge to the center of the lesion, (F) pathological image of the same site as the photoacoustic image, and (G) rectum, Type2, pT3

In submucosal invasive cancers, thick and prominent vascular networks were visible in the superficial layer of esophageal cancers, the most invaded area of gastric cancers, and both the superficial and most invaded regions of colorectal cancers (Figure 5). The number of blood vessels recognized inside the lesions was much smaller than that along the surface and in the invaded area of the lesions in the esophageal, gastric, and colorectal specimens. The vessel findings of T2–4 cancers were similar to those of submucosal invasive cancers (Figure 6).

**FIGURE 7:**
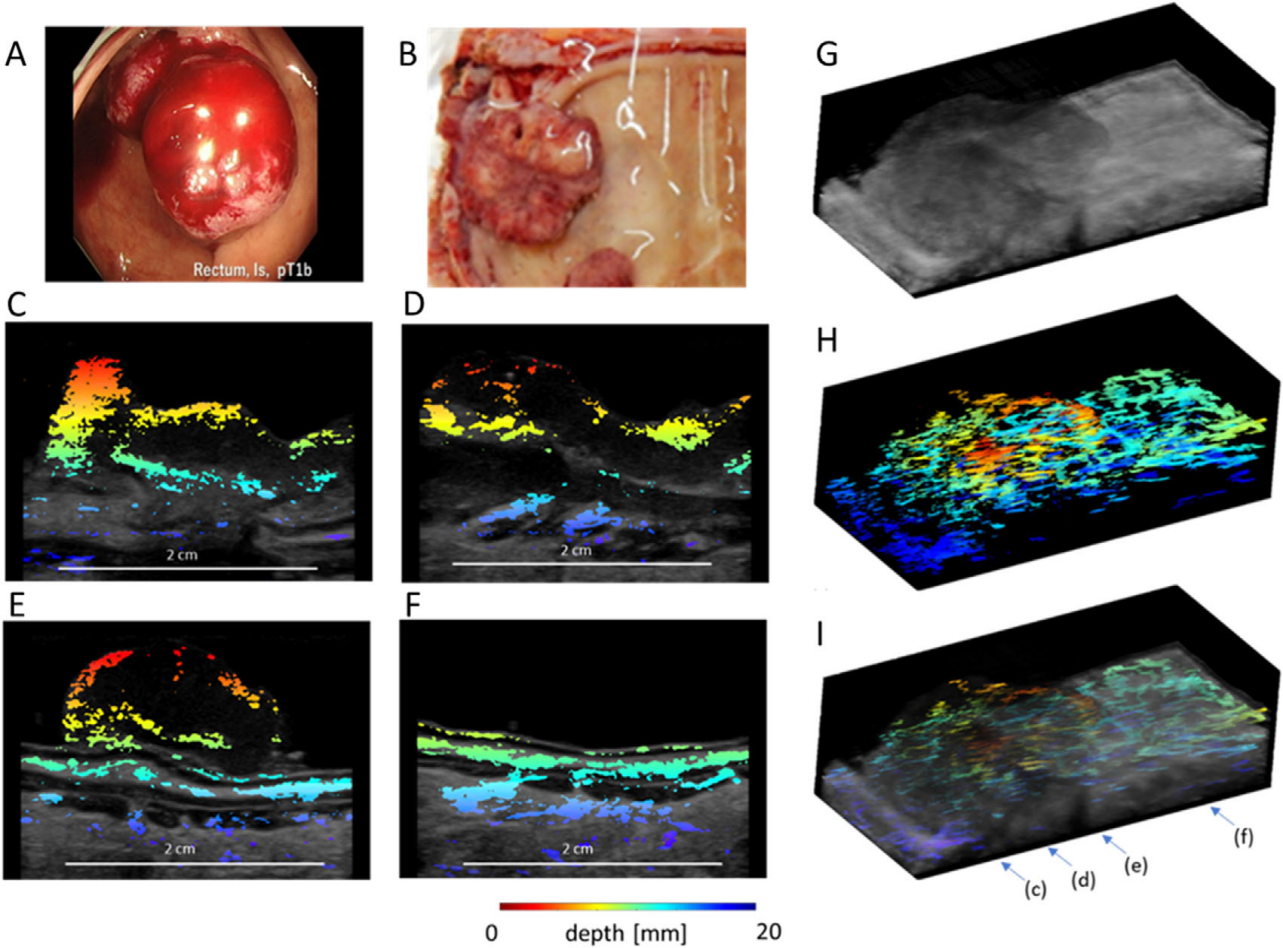
The 3D photoacoustic (PA) image superimposed on a 3D (ultrasound) US image of rectal T1 lesion. The endoscopic image of rectal T1 cancer, (A, B) the photograph of the resected specimen in the PA image acquisition, (C–F) the 2D cross‐sectional PA images superimposed on the gray‐scaled US images, (G) the 3D US and (H) PA images of the specimen, and (I) the 3D PA image superimposed on the 3D US image. The arrows with (I) indicate the positions of the cross‐sections for (C–F). The color bar indicates the depth from the surface of the probe

Figure 7 shows a series of PA images and 3D construction of a case of rectal T1 cancer. The surface, peripheral, and deep blood vessels of the lesion can be easily recognized but not the central blood vessel of the lesion. In addition, penetrating branches from the muscular layer to the submucosal layer are also well visualized outside the lesion (Supplement Video 1).

**FIGURE 8:**
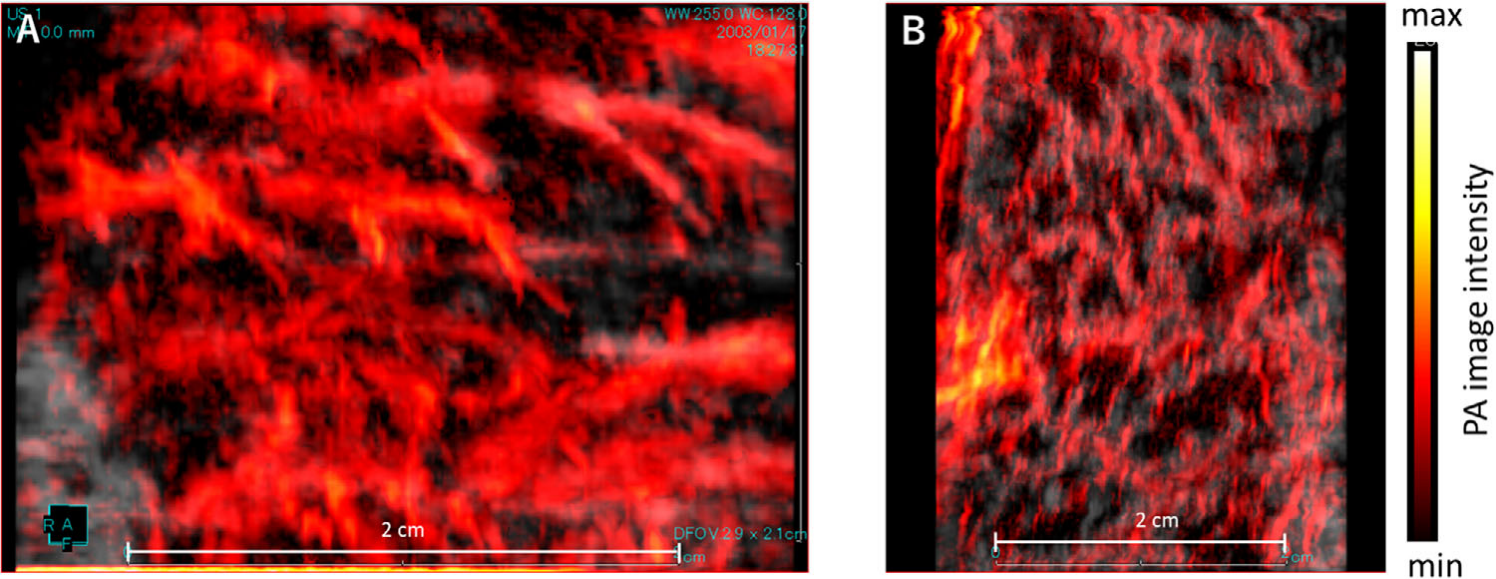
Maximum intensity projections of the photoacoustic (PA) images of normal gastric and rectal walls of a living pig. PA images are superimposed on gray‐scaled B‐mode ultrasound (US) images in (A) and (B). The color bars indicate the PA image intensity. (A) Gastric wall and (B) rectal wall

In the images of living pigs, it was possible to image the vascular network deeper than the submucosa in both the stomach and large intestine (Figure 8).

## DISCUSSION

This study illuminated the potential of PA visualization of deep vessels using specimens from human GI cancer.

Diagnoses of GI cancer are typically made morphologically. On the other hand, when making a diagnosis of early cancer that involves the selection of treatment (endoscopic treatment or surgical treatment), the following procedures are performed in addition to morphological diagnosis: chromoendoscopy, vascular observation using IEE, pit observation using magnifying endoscopy, and cellular level observation using a 500‐fold magnifying endoscopy. However, none of these methods lead to an accurate diagnosis as they only assume the condition of the deep parts based on the information from the lesion surface. While EUS and OCT enable tomographic imaging, their resolution and depth of measurement are limited. Hence, diagnostic endoscopic devices that can obtain information on deep parts need to be developed. As for the deeper layer information, detecting vessel networks in GI walls may help achieve not only an accurate diagnosis of the depth of invasion but may also be used to prevent hemorrhage during endoscopy, predict the effect of chemotherapy, and evaluate drug delivery. PA imaging has the potential to overcome the limitation.

Extirpated specimens were used in this study because no devices for endoscopic measurement were available. The PA/US echo imaging system was developed jointly by Ishihara et al. and Fujifilm Co, and an extracorporeal probe was used for the measurements.^13^, ^30^, ^31^ It was reported that our PA/US echo imaging system provided 0.3 mm spatial resolution in the phantom experiment with the tubes containing the bovine blood at depths up to 20 mm by mimicking the blood vessels, and the system detected the vessel‐mimicking wire with a diameter smaller than 30 μm.^30^ The system performance allowed us to image the micro‐vessels in the lesions and penetrating branches in the deeper layer. PA imaging has higher sensitivity than the US power Doppler imaging. By using the PA/US echo system with the transrectal probe, Horiguchi and colleagues demonstrated the PA image captured the angiogenic micro‐vessels of prostate cancer that had a diameter of only 10–50 μm^13^ and could not be detected by the power Doppler imaging.^34^ Based on the previous and this studies, it is anticipated that PA imaging detects the vessels of GI lesions in vivo. On the other hand, it is difficult to differentiate between Doppler signal and background disturbance, because the intratumor vessels are small with slow flow rate.^35^ The most salient advantage of this system is that it can identify the layer of the wall to which the vessel belongs because it allows overlapping of US and PA images. In addition, as shown in Figure 6, clearer images can be obtained by 3D imaging.

Separate evaluations of the esophagus, stomach, and colorectal cancer suggested that there may be differences in deep vessel networks between organs. While no differences in vessel networks were observed between normal wall specimens, vessels were observed in the following different parts in the case of cancer lesions: the surface of the esophagus, the deepest part of the stomach, and the surface and deepest part of the colon. Particularly, vessels can be observed in the deepest parts of the stomach and colon lesions; therefore, using PA imaging in combination with US echo imaging may help diagnose the depth of invasion. Clear images of the layer structure and vessels were obtained for specimens obtained through surgical resection. However, images of specimens obtained through endoscopic resection could not be evaluated because their resection surfaces were damaged by bleeding and cauterization. Because of this, early cancer evaluation, which is the most important procedure in diagnosing the depth of invasion, could not be performed. This issue needs to be addressed in a future study.

When compared with images that have been reported in existing animal experiments, dotted vessels were observed on 3D‐constructed images.^30^, ^31^ It was considered that there was no blood flow due to the extirpated specimens, and the vessels reflected Hb in congestive venous blood. In order to validate that it was Hb, healthy in vivo porcine mucosa was used for real‐time measurement. As shown in Figure 8, it was confirmed that the dotted vessels reflected Hb, with clearly drawn vessel networks both in the stomach and the colon.

Although several probes that allow endoscopic measurement have thus far been reported only at a basic research level, no such probes are currently available for clinical use.^25^, ^36^, ^37^ If such a probe is integrated into an endoscopic device, the visualization of the vascular network in deeper layers of the GI walls may be possible during endoscopy. Therefore, it is hoped that PA imaging as a functional diagnostic endoscope will improve diagnostics.

This study has several limitations. First, this study examined extirpated lesions and did not perform in vivo measurements. Future studies should involve pathological examination through such means as immunostaining. It is yet to be clarified how much of the blood vessel diameter a PA image can visualize, and it is anticipated that there is a difference in vessel diameter between in vivo and resected specimens. Therefore, this study was based on vascular images of the GI wall of living pigs. Second, this study examined a small number of cases and did not examine specimens with different histological or differentiation type lesions. Therefore, a larger number of cases need to be examined.

Our study concluded that the deep vessel networks of neoplastic GI lesions may be visible on PA imaging. Furthermore, we observed variation in deep vessel networks among invasive and noninvasive cancers and each GI organ.

## CONFLICT OF INTEREST

Author H.l. is an Associate Editor of DEN Open. Other authors declare no Conflict of lnterests for this article.

## Supporting information


**Supplement Video 1**. 3D photoacoustic image of the rectal lesion. The endoscopic image of the rectal lesion, 20 slices of the tomographic 2D PA images superimposed on the US images with color bars indicating PA image intensities, and the rotating 3D PA image merged with 3D US image of the surgically resected rectal lesion constructed from the 2D PA and US images are presented in sequence.
